# Detection of Endotoxin Contamination of Graphene Based Materials Using the TNF-α Expression Test and Guidelines for Endotoxin-Free Graphene Oxide Production

**DOI:** 10.1371/journal.pone.0166816

**Published:** 2016-11-23

**Authors:** Sourav P. Mukherjee, Neus Lozano, Melanie Kucki, Antonio E. Del Rio-Castillo, Leon Newman, Ester Vázquez, Kostas Kostarelos, Peter Wick, Bengt Fadeel

**Affiliations:** 1 Nanosafety & Nanomedicine Laboratory, Division of Molecular Toxicology, Institute of Environmental Medicine, Karolinska Institutet, Stockholm, Sweden; 2 Nanomedicine Laboratory, Faculty of Medical & Human Sciences and National Graphene Institute, University of Manchester, Manchester, United Kingdom; 3 Particles-Biology Interactions Laboratory, Swiss Federal Laboratories for Materials Science and Technology, St. Gallen, Switzerland; 4 Department of Organic Chemistry, University of Castilla-La Mancha, Ciudad Real, Spain; INSERM, FRANCE

## Abstract

Nanomaterials may be contaminated with bacterial endotoxin during production and handling, which may confound toxicological testing of these materials, not least when assessing for immunotoxicity. In the present study, we evaluated the conventional Limulus amebocyte lysate (LAL) assay for endotoxin detection in graphene based material (GBM) samples, including graphene oxide (GO) and few-layered graphene (FLG). Our results showed that some GO samples interfered with various formats of the LAL assay. To overcome this problem, we developed a TNF-α expression test (TET) using primary human monocyte-derived macrophages incubated in the presence or absence of the endotoxin inhibitor, polymyxin B sulfate, and found that this assay, performed with non-cytotoxic doses of the GBM samples, enabled unequivocal detection of endotoxin with a sensitivity that is comparable to the LAL assay. FLG also triggered TNF-α production in the presence of the LPS inhibitor, pointing to an intrinsic pro-inflammatory effect. Finally, we present guidelines for the preparation of endotoxin-free GO, validated by using the TET.

## Introduction

Carbonaceous nanomaterials, including carbon nanotubes (CNTs), and graphene based materials (GBMs) such as graphene oxide (GO), hold significant promise in engineering and medicine due to their intrinsic electro-mechanical properties [1, 2]. However, for the successful development and application of these materials, a comprehensive study of their potential toxicity is required [3, 4]. In particular, it is important to determine whether any nanomaterial effects on immune-competent cells such as macrophages or dendritic cells occur and whether these are due to intrinsic properties of the nanomaterials or whether they may be caused, for instance, by endotoxin contamination [5, 6].

Endotoxins, also known as lipopolysaccharides (LPS), are large (molecular weight: 200 to 1000 kDa), heat-stable molecules that form part of the outer membrane of gram-negative bacteria [7]. They are the most common contaminants of water systems and biomaterials and are resistant to conventional methods of sterilization. LPS is composed of three parts: the proximal hydrophobic lipid A region which anchors LPS to the outer leaflet of the outer membrane of bacteria, the distal hydrophilic O-antigen repeats which extend into the surrounding aqueous medium, and the interconnecting core oligosaccharide [8]. LPS is a potent inflammatory mediator which activates immune cells via Toll-like receptors (TLRs) leading to the secretion of pro-inflammatory mediators, e.g., tumor necrosis factor (TNF)-α, and interleukin (IL)-1β [9, 10]. Exposure of humans to endotoxin may results in septic shock and organ failure. Therefore, according to US Food and Drug Administration (FDA) guidelines (June 2012) the endotoxin limit is 0.5 EU/mL or 20 EU/device for products that directly or indirectly contact the cardiovascular or lymphatic system [11].

**Fig 1:**
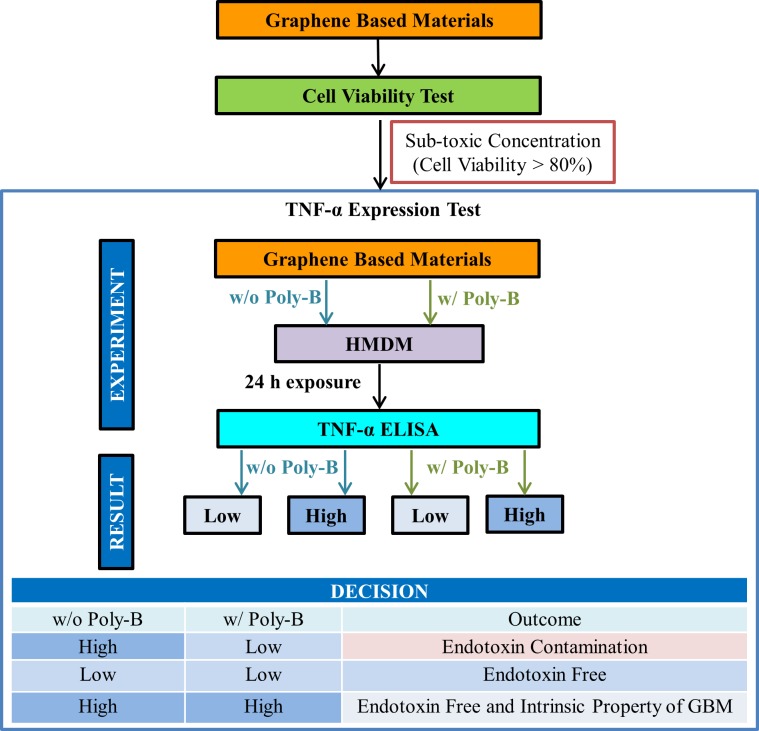
The TNF-α Expression Test (TET) for endotoxin detection in GBMs.

Endotoxin detection in pharmaceutical products is performed using two different methods. The *in vivo* rabbit pyrogen test (RPT) enables the detection of pyrogens in general by measurement of possible fever development after injection of the test sample [12]. The second type of endotoxin detection method, the Limulus amebocyte lysate (LAL) assay is based on the blood of wild horseshoe crab populations. While the RPT assay can only detect the presence of endotoxins indirectly, the LAL assay is more specific to endotoxins as it takes advantage of the LPS-sensitive serine protease Factor C. Upon activation, Factor C induces a coagulation cascade leading to the amplification of the LPS stimulus and the formation of a firm gel clot. All LAL assays are in principle based on this coagulation cascade, but they have been further modified to enable quantitative determination of endotoxins. Today, three LAL assay formats with different read-out are available: gel-clot (semi-quantitative), turbidimetric, and chromogenic (quantitative) [13]. The RPT is an expensive method which requires a large number of animals and also yields large variations in test performance, but is still used for assessment of pyrogenicity of a majority of biological products including blood products and vaccines owing to interference when using the LAL test. For more than 30 years, FDA has accepted the use of the LAL test for endotoxins instead of the RPT. More recently, the recombinant factor C (rFC) assay and the monocyte activation test (MAT) were recognized as alternatives to the LAL assay [11]. The MAT, which mimics the human fever reaction, was established as an alternative test for pyrogen testing [14], and implemented into the European Pharmacopoeia (Monograph 2.6.30) in 2009 as an alternative to the RPT. Importantly, the European Directive 2010/63/EU on the protection of animals used for scientific purposes enforces the replacement of animal tests when validated alternatives exist. While the LAL assay is known to be very sensitive, several laboratories have reported problems of interference of nanoparticles with one or more of the LAL assay formats [12, 15–18]. Previous studies have suggested that TLR4 reporter cells could be used to evaluate endotoxin contamination of metal/metal oxide nanoparticles [17]. There is limited information available on whether GBMs interfere with commonly used endotoxin assays. On the other hand, recent work has implied that GO could trigger cell death via TLR4 [19], meaning that the use of TLR4 reporter cells would yield ambiguous results. Here, we compared different LAL assays formats and found that some graphene oxides (GO) interfere with this commonly used assay. To circumvent this problem, we devised the TNF-α expression test (TET) using primary human monocyte-derived macrophages (HMDM) to detect LPS contamination in GBMs (Fig 1). We also describe procedures for sterile synthesis of GO.

## Materials and Methods

### Reagents

The endpoint chromogenic LAL test kit and the LAL Gel clot assay kit were purchased from Lonza (Basel, Switzerland), and the Endosafe®-PTS™ cartridges were purchased from Charles River, (Charleston, SC). LPS and the LPS inhibitor, Polymyxin B sulfate (CAS 1405-20-5) were both obtained from Sigma-Aldrich (Stockholm, Sweden). CD14 MicroBeads were purchased from Miltenyi Biotec (Bergisch Gladbach, Germany). Lymphoprep™ was obtained from Axis-Shield (Oslo, Norway). RPMI-1640 cell culture medium, L-glutamine, penicillin, streptomycin and fetal bovine serum (FBS) were purchased from Gibco Invitrogen Corporation (Paisley, UK). Recombinant human macrophage colony-stimulating factor (M-CSF) was purchased from Novakemi (Handen, Sweden). The AlamarBlue® reagent was from Invitrogen (Stockholm, Sweden).

### Synthesis of graphene based materials

Five GBMs were studied, i.e., four graphene oxide (GO) samples and one few-layered graphene sample (FLG). All GO samples were prepared by the modified Hummer’s method as previously described [20]. Briefly, two GO samples (designated here as GO-A and GO-B) were synthesized in-house from precursor graphite flakes (Graflake 9580 from Nacional Grafite Ltd., Brasil) which were mixed with sodium nitrate in a round bottom flask, and then sulfuric acid was added slowly to the mixture. After obtaining a homogenized mixture, potassium permanganate was slowly added and the mixture was maintained for 30 min. Water was added drop-wise due to the violent exothermic reaction and the temperature was continuously monitored and maintained at 98°C for 30 min. The mixture was further diluted in water and hydrogen peroxide was added for the reduction of the residual potassium permanganate, manganese dioxide and manganese heptoxide to soluble manganese sulfate salts. The resulting mixture was purified by several centrifugation steps until a viscous orange/brown gel-like layer of pure GO started appearing on top of the oxidation by products at neutral pH. This GO-gel like layer was extracted carefully using warm water. Final concentrations ranged between 1 and 2 mg/mL were obtained with a yield of ca. 10%. The sample was denoted GO-A. The GO sample produced after validating the endotoxin-free conditions was denoted GO-B. In addition, a commercially available GO (designated hereafter as GO-C) was supplied by Antolin Group (Burgos, Spain) and was produced from carbon fibres, GANF^®^, by a modified Hummer’s method [21, 22]. Another commercially available GO, designated here as GO-D, was supplied by Graphenea (San Sebastian, Spain). Finally, FLG was produced in-house by exfoliation using the ball-milling procedure and was lyophilized, leading to a black powder which was readily dissolved in culture media (Vazquez et al., details to be published elsewhere).

### Material characterization

The morphology and lateral dimensions of GO sheets prepared by the modified Hummer’s method (i.e., GO-A, GO-B, GO-C and GO-D), as well the FLG synthesized by the ball-milling method, were studied by transmission electron microscopy (TEM) and optical microscopy. Surface properties were determined using by Raman spectroscopy and electrophoretic mobility measurements, the degree of functionalization was measured using thermogravimetric analysis (TGA), and the chemical composition and C:O ratio by X-ray photoelectron spectroscopy (XPS). *Transmission Electron Microscopy*. TEM was performed to measure the average size and morphology of GO sheets using a BioTwin electron microscope (Philips/FEI), Tecnai 12 instrument operated at 100 keV and using JEOL 2100F TEM/STEM electron microscope operating at 200 kV. Twenty μL of 200 μg/mL of GBM was placed on a carbon-coated copper grid (400 mesh). Filter paper was used to remove the excess of material. *Optical microscopy*. Bright field microscopy using a Zeiss Primovert microscope was used to measure the average lateral dimension. *Raman spectroscopy*. Raman spectra were recorded for GBMs (20 μL of 100 μg/mL) that were drop casted onto glass and left to dry at 37°C for 2 h. Measurements were carried out at 50x magnification and laser excitation wavelength of 532 nm and 633 nm at a power of 0.4 mW using an Invia Renishaw micro-Raman spectrometer and a micro-Raman spectrometer (Thermo Scientific, UK), respectively. An average of five different locations within each sample was measured to calculate the ID/IG ratio. *ζ-potential measurements*. Electrophoretic mobility (μ) was measured by Malvern Zetasizer Nano ZS (UK) after dilution of samples with water in disposable Zetasizer cuvettes (Malvern Instruments). Default instrument settings and automatic analysis were used for all measurements, where the μ was converted automatically by the equipment software to zeta potential (ζ) values as it is directly related to zeta potential by Henry’s equation. All values for samples prepared are triplicate measurements, values were mean ± SD. *Thermogravimetric analysis*. The weight loss measurements of GBM samples were performed by TGA using a Pyris 6 (Perkin-Elmer Ltd.) and TGA Q50 (TA Instruments). Hence, 1–2 mg of GBM weighed into a ceramic crucible was analyzed from 25 to 995°C at 10°C/ min with a nitrogen flow of 20 mL/min. *X-ray photoelectron spectroscopy*. The chemical composition of GBM sheets was studied by XPS at NEXUS facility (the UK's National EPSRC XPS Users' Service, hosted by nanoLAB in Newcastle-upon-Tyne). XPS was recorded using a Thermo Theta Probe XPS spectrometer with a monochromatic Al K-α source of 1486.68 eV. The survey XPS spectra were acquired with pass energy (PE) of 200 eV, 1 eV step size, 50 ms dwell time and averaged over 5 scans. The etching was 90 seconds. The high resolution C1s XPS spectra were acquired with PE of 40 eV, 0.1eV step size, 100 ms dwell time and averaged over 20 scans. Spectra from insulating samples have been charge corrected by shifting all peaks to the adventitious carbon C 1s spectral component binding energy set to 284.6 eV. CasaXPS software was used to process the spectra acquired at NEXUS. Elemental analysis was performed using the analyzer LECO CHNS-932 (Model NO: 601-800-500).

### Limulus amebocyte lysate (LAL) assay

Three different formats of the LAL assay, i.e., Endpoint Chromogenic LAL assay, Endosafe®-PTS™-assay, and Gel Clot assay, were used in this study. All GBMs used in these assays were prepared in endotoxin-free distilled water to avoid any external interference in the assay except by the material itself. The *Endpoint Chromogenic LAL Assay* was performed according to the manufacturer’s guidelines with an assay sensitivity of 1.0–0.1 EU/ml. In brief, the GBM samples, at 50 μg/ml, were mixed with the LAL supplied in the test kit and incubated at 37°C for 10 min. A peptide substrate solution was then mixed with the LAL-sample mixture and incubated at 37°C for an additional 6 min. The reaction was then stopped with a stop reagent provided with the kit. In addition to the complete reaction mixture, i.e., GBM + LAL + substrate, two additional mixtures were prepared to check the possible interference of GBM in the assay, namely GBM + LAL and GBM + substrate. If endotoxin is present in the sample, a yellow color should develop only in the complete reaction mixture, not in the latter two mixtures. The absorbance of the enzymatically cleaved p-nitroaniline part of the substrate peptide was measured at 405 nm in a Tecan Infinite F200 plate reader (Stockholm, Sweden). Since this absorbance is in direct proportion to the amount of endotoxin present, the concentration of endotoxin can be calculated from a standard curve using LPS. The *Endosafe®-PTS™-Assay* was performed according to the manufacturer’s instructions by application of PTS cartridges with an assay sensitivity of 1.0–0.01 EU/ml. Cartridges included control standard endotoxin (CSE) as positive control. GOs were diluted in LAL-reagent water in a two-fold dilution series. The cartridges contained two sample channels for endotoxin detection as well as two channels with internal endotoxin spike. Test results obtained by individual cartridges were accepted as valid if three acceptance criteria were fulfilled: a spike recovery within a range of 50–200%, a sample coefficient of variation below 25%, and a spike coefficient of variation below 25%. In case spike recovery was beyond the valid range, the sample was further diluted in LAL-reagent water. The *LAL Gel Clot Assay* was performed as described in [23]. The assay sensitivity of 0.03 EU/ml was confirmed for all kits applied. Assay performance was controlled by the application of a CSE from *Escherichia coli* O55:B5, as well as endotoxin-free LAL-reagent water (negative control).

### Isolation of primary macrophages

Peripheral blood mononuclear cells (PBMC) were isolated from buffy coats obtained from healthy human blood donors (Karolinska University Hospital, Stockholm, Sweden) by density gradient centrifugation using Lymphoprep™, as described previously [24]. Then, the PBMCs were positively selected for CD14 expression using CD14 MicroBeads. To obtain human monocyte-derived macrophages (HMDM), CD14+ monocytes were cultured in RPMI-1640 cell medium supplemented with 2 mM L-glutamine, 100 IU/mL penicillin, 100 μg/mL streptomycin, and 10% heat inactivated FBS, supplemented with 50 ng/mL recombinant M-CSF for three days in 96 well plates.

### Ethics statement on human samples

As mentioned above, cells were isolated from buffy coats obtained from healthy adult blood donors at the Karolinska University Hospital, Stockholm, Sweden. The donors are approved and covered by insurance according to the regulations at the Karolinska University Hospital. These buffy coats contain white blood cells and are considered a waste product after the red blood cells have been utilized for blood transfusions. The identity of the blood donors is unknown to the scientists performing the experiments. We were previously notified by the Ethical Committee for Human Studies in Stockholm that no specific ethical permit is required for *in vitro* (cell culture) studies of nanomaterials on cells derived from human buffy coats, such as the studies reported herein, since the data cannot be traced back to the individual blood donors (see 2006/900-31/3; decision 2006/3:8).

### Alamar blue cell viability assay

HMDMs were exposed to GBMs up to 75 μg/ml in RPMI-1640 medium supplemented with 10% FBS (without M-CSF) in parallel to 5% DMSO as a positive control for cell death for 24 h. Then, the Alamar Blue (AB) assay was performed according to the manufacturer’s instruction. Briefly, medium was removed, cells were rinsed with PBS and 100 μl of AB medium (5% [v/v] solution of AlamarBlue® reagent), prepared freshly in RPMI-1640 medium, were added to each well. After 2 h of incubation at 37°C, fluorescence was measured at the respective excitation and emission wavelength of 531 nm and 595 nm using a Tecan Infinite F200 plate reader. AB alone and cell culture medium alone were included as blanks. The experiment was performed with at least three biological replicates and six technical replicates for each concentration of GBM. Results were expressed as % cell viability *versus* control. Potential interference with the assay was evaluated in an acellular system by incubating 75 μg/mL of each of the GBMs with the AB reagent for 2 h at 37°C.

### TNF-α measurement by ELISA

HMDMs were incubated with GBMs at 25 μg/ml or 50 μg/ml concentrations in the presence or absence of polymyxin B sulfate (10 μM) for 24 h in complete RPMI medium supplemented with 10% FBS. Polymyxin B sulfate is a potent antibiotic that interacts with the lipid A region of LPS and thereby neutralizes its activity [25]. LPS (100 ng/ml) was included as a positive control. Following exposure, cell culture supernatants were collected and the secretion of TNF-α was determined by ELISA according to the manufacturer’s instruction (MABTECH, Nacka Strand, Sweden). The assays were performed in macrophages derived from four different human donors (i.e., four biological replicates) and at least three technical replicates were used for cells of each donor. A standard curve was generated based on LPS-induced TNF-α expression by HMDM. This allowed for the quantification of LPS present in the GBM samples. The difference between the TNF-α expression induced by GBMs with or without Poly-B corresponds to the endotoxin present in the sample.

### Statistics

Data are expressed as mean values ± standard deviation (SD). Changes in the variables (e.g., cells isolated from different human blood donors) for different assays were measured using Student’s t-test, and differences among mean values were considered significant when p-values are ≤ 0.05, ≤ 0.01 or, ≤ 0.001. One-way Anova with post-hoc Turkey’s test between groups with and without polymyxin-B sulfate was performed in the TET assay. For cell viability (i.e., Alamar blue assay) and endotoxin detection using TNF-α expression by HMDM, at least three or more donors (n) were used.

## Results and Discussion

### GBM synthesis and characterization

The GBMs (GOs and FLG) were subjected to rigorous physicochemical characterization. TEM analysis indicated that the average lateral dimensions of GO-A, GO-B, GO-C, GO-D and FLG were 8±5 μm, 10±8 μm, 85±50 nm, 0.8–7μm, and 630±390 nm, respectively and all the GBMs were found to be single-few layers thick (Table 1). Raman spectrometric analysis showed the D and G bands for all the GOs were around 1345 cm-1 and 1580 cm-1, while the D and G band ratios are presented in Table 1. TGA indicated that 39%, 41%, 52%, 47%, and 7% of GO-A, GO-B, GO-C, GO-D and FLG were functionalized (Table 1). Furthermore, the detailed chemical composition of the five GBMs, including oxygen content, contributed by different functional groups such as carboxylic, carbonyl and epoxide, measured by elemental analysis and XPS, is also presented in Table 1.

**Table 1:** Physicochemical characterization of GBMs.

*Physicochemical properties*	*Technique*	*GO-A*	*GO-B*	*GO-C*	*GO-D*	*FLG*
***Lateral dimension***	*TEM*	10–15 μm	15–20 μm	85 ± 50 nm	0.8–7 μm	630 ± 390 nm
***Thickness***	*TEM*	1–2 layers	1–2 layers	1 to Few layers	1–2 layers	Few Layers
***Surface charge***	*ζ-Potential*	-52.5 ± 1.8 mV	-50.4 ± 3.0 mV	-36.36 ± 0.7 mV	-40.6 ± 1.5 mV	-16 ± 0.01 mV
***Functionalization degree***	*TGA*	39%	41%	52%	47%	7%
***Chemical composition***	*Elemental Analysis and XPS*	C: 70.9%, O: 29.1%	C: 68.8%, N: 0.4%, O: 30.8%	C: 45.9 wt%, H: 3.2 wt%, N: 0.23 wt%, S: 0.34 wt%, O: 50.2 wt%	C: 66.9%, O: 31.0%, S: 1.9%, B: 0.2%	C: 94.3 wt%, H: 0.42 wt%, N: 0.36 wt%, O: 4.92 wt%
***C*:*O ratio***	*XPS*	2.4	2.2	0.9	2.2	9.4
***Degree of defects (I*_*D*_*/I*_*G*_*)***	*Raman Spectroscopy*	1.35 ± 0.01	1.30 ± 0.04	0.82 ± 0.02	1.33 ± 0.03	0.5 ± 0.1

### Endotoxin testing by conventional LAL assays

**Fig 2:**
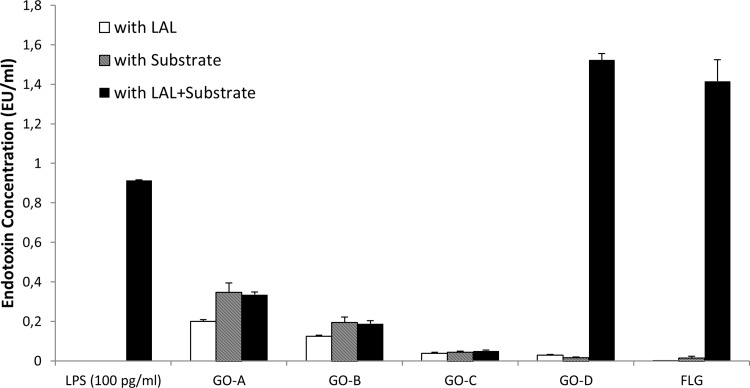
Endotoxin detection in GBMs using the endpoint chromogenic LAL assay. GBMs (50 μg/ml) were incubated with LAL (containing enzyme), or substrate, or both LAL and substrate. After the incubation period, the absorbance of the substrate was measured. The data showed significant LPS contamination in GO-D and FLG, while assay interference was observed in the case of GO-A and GO-B.

Following synthesis/procurement and characterization of the GBM samples, their endotoxin levels were measured using standard methods, i.e., different formats of the LAL assay (see Methods). The endpoint chromogenic LAL assay results showed similar absorbance in cases of GO-A and GO-B with or without the presence of LAL and substrate (Fig 2). This indicated false positive results for these GO samples, due to optical interference with the LAL assay at the detection wavelength. GO-C displayed very low endotoxin levels in the endpoint chromogenic LAL assay (Fig 2) and it could therefore not be concluded whether or not there is any interference with the assay. On the other hand, GO-D and FLG displayed high levels of endotoxin in this assay, i.e., 1.5 EU/ml and 1.4 EU/ml, respectively, and low intrinsic material absorbance at the wavelength used (405 nm).

The Endosafe®-PTS™ system is a portable and easy-to-use endotoxin test device which allows rapid detection of endotoxins in aqueous media. Endotoxin detection is performed by a chromogenic LAL assay contained in single-use cartridges already supplied with internal CSE for verification of the detection process. We found significant interference for GO-A at a concentration of 62.5 μg/ml and 31.25 μg/ml (Table 2). The test results were not valid due to either enhancement of the assay with spike recovery rates above 200% or inconsistent results of the channels within the cartridges. Further two-fold dilution to 15.625 μg/ml led to endotoxin values between 0.05 EU/ml and no detectable endotoxin (<0.01 EU/ml). Lower rate of interference was observed for GO-B (Table 2). At three different concentrations (31.25, 15.625 and 7.8 μg/ml) no endotoxin could be detected. Spike recovery values were always higher than 150% pointing to assay enhancement by the material, most likely due to the intrinsic absorbance of GO. For GO-C, the mean endotoxin value determined was 0.03 EU/ml at a concentration of 31.25 μg/ml GO (Table 2). GO-D showed non-valid enhancement at a concentration of 15.625 μg GO/ml and higher, similar to GO-A, at 31.25 μ/ml which indicated significant interference. At a concentration of 7.8 μg/ml, GO-D was endotoxin-negative with endotoxin values below the detection limit of < 0.01 EU/ml.

**Table 2:** Endotoxin screening of GO by using Endosafe®-PTS™.

Samples	Concentration in μg/ml	Mean calculated endotoxin concentration in EU/ml	
		Pyrogent™ Plus LAL Gel Clot	Endosafe®-PTS™ kinetic chromogenic LAL
GO-A	62.5	0.24	Non-valid enhancement
	31.25	0.12	Non-valid enhancement
	15.6	0.06	< 0.01 to 0.05
GO-B	62.5	0.06	< 0.01
	31.25	0.03	< 0.01
	15.6	< 0.03	< 0.01
GO-C	31.25	0.06	0.03
	15.6	0.03	< 0.01
	7.8	< 0.03	< 0.01
GO-D	31.25	0.48	Non-valid enhancement
	15.6	0.24	Non-valid enhancement
	7.8	0.12	< 0.01

The LAL gel clot assay is the original LAL assay format, introduced in the 1970s, and enables semi-quantitative determination of endotoxin. It is recommended to be applied for clarification in case of a discrepancy between results of different LAL assay formats as it is regarded as being the most robust and least prone to interference, as optical interference can be ruled out, since the detection of endotoxins is based on visual inspection of gel clot formation. Nevertheless, we previously noted that the LAL gel clot assay showed significant interference with iron oxide-silica-core shell nanoparticles as well as other types of nanoparticles, leading either to assay enhancement or inhibition [26]. The GO dispersions were found to be endotoxin positive in the LAL gel clot assay at concentrations as low as 7.8 μg/ml (GO-A), 31.25 μg/ml (GO-B), 15.625 μg/ml (GO-C), and 1.95 μg/ml (GO-D), respectively (Table 3). However, as pointed out, the gel clot assay is not quantitative.

**Table 3:** Endotoxin screening of GO by LAL gel clot assay.

Samples	Sample concentration in μg/ml							Controls		Calculated EU/ml for concentration of 50 μg/ml GBM
	62.5	31.25	15.6	7.8	3.9	1.95	0.98	Negative control LAL reagent water	Positive control CSE E.coli 0.06 EU/ml	
GO-A	+	+	+	+	-	-	-	-	+	0.19
GO-B	+	+	-	-	-	-	-	-	+	0.05
GO-C	+	+	+	-	-	-	-	-	+	0.10
GO-D	+	+	+	+	+	+	-	-	+	0.77

Note: + sign is used to depict an endotoxin positive signal (i.e., gel clot formation) observed for the respective concentrations of GO, while–sign is used for GO concentrations where the endotoxin levels were below the threshold level of detection, i.e., 0.03 EU/ml and therefore did not form any clots in the assay.

### The TNF-α Expression Test (TET)

**Fig 3:**
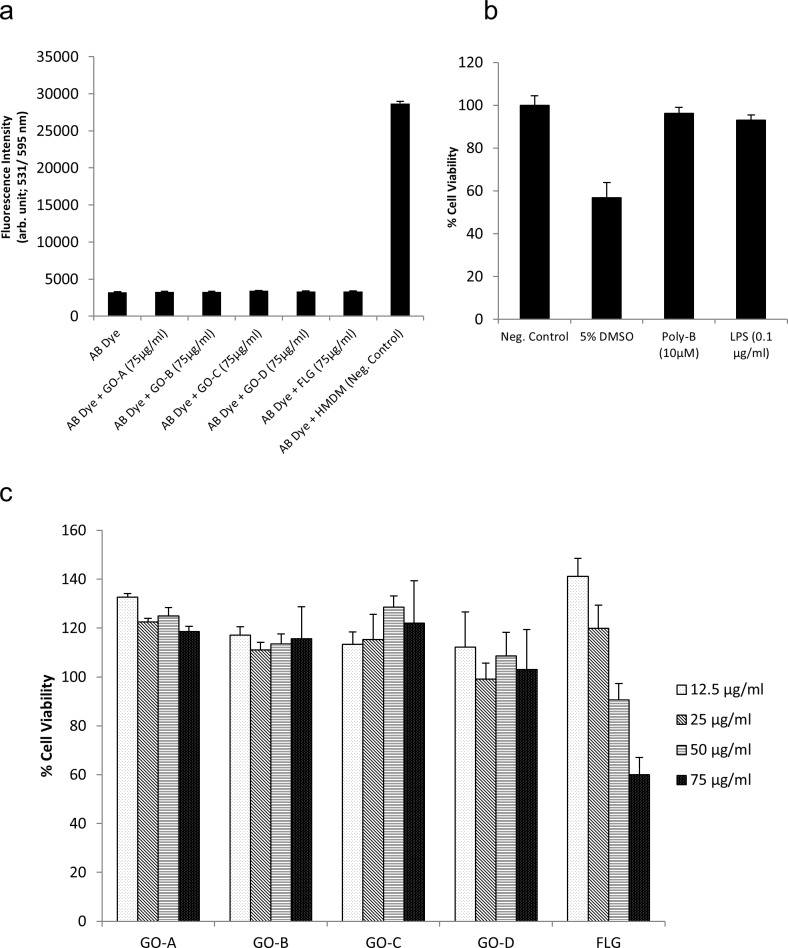
Cell viability assessment of primary human monocyte-derived macrophages (HMDM) following GBM exposure. a) No interference in the Alamar Blue cell viability assay of any of the GBMs tested. b) No toxicity was observed for LPS (0.1 μg/ml), or the LPS inhibitor, polymyxin B sulfate (Poly-B, 10 μM) while 5% DMSO triggered cell death as expected (positive control). c) Cell viability was evaluated by Alamar Blue assay in HMDM exposed to different concentrations of GO-A, GO-B, GO-C, GO-D, and FLG for 24 h. Note the dose-dependent cytotoxicity of FLG. The experiment was conducted with at least three independent donors per sample. The results are expressed as mean percentage change relative to unexposed control ± S.D. The control values were set at 100% cell viability. Statistical analyses were carried out using the paired Student's t-test in Microsoft® Excel, where 95% significance levels were accepted. (* = p ≤ 0.05, ** = p ≤ 0.01, *** = p ≤ 0.001).

The MAT is used as an alternative to the LAL assay, and Dobrovolskaia et al. recently reported that this assay could be used to resolve discrepancies arising from different LAL tests when assessing nanomedicines [27]. The MAT is based on the use of a human monocytic cell line, MM6, and ELISA-based detection of IL-6 in the cell culture supernatants. However, while monocytic cell lines, such as MM6 and THP.1 are sensitive to LPS, it is known that undifferentiated monocytes produce lower level of inflammatory markers (e.g., TNF-α), than their differentiated counterparts upon exposure to LPS [28]. Therefore, to establish a sensitive, quantitative method for the assessment of endotoxin content in GBM samples, and in order to distinguish between material-intrinsic effects *versus* effects arising from endotoxin contamination, we set up the TNF-α expression test (TET) using primary human macrophages as our model system (Fig 1). The TET is based on the detection of TNF-α in the presence or absence of the specific LPS inhibitor, polymyxin B sulfate. However, in order to use HMDM for endotoxin testing, a non-toxic dose of the test material should be established, since cytokine production may not be reliably evaluated at cytotoxic doses of the test compound. To this end, following differentiation of primary human monocytes into macrophages for three days, cells were exposed to different concentrations of GO for 24 h and cell viability was determined using the Alamar Blue assay. No interference for any of the GBMs at the highest concentration used, i.e., 75 μg/ml, was observed in this assay (Fig 3A). The positive control used in this assay, i.e., 5% DMSO, showed ~55% cell viability after 24 h, while LPS and polymyxin B sulfate did not trigger any significant loss of cell viability (Fig 3B). As seen in Fig 3C, no cytotoxicity was observed after 24 h exposure of HMDM up to 75 μg/ml concentration for any of the GBM samples tested. In fact, an apparent increase in cell viability was observed (i.e., values higher than control) (Fig 3C). The reason for this increase in the Alamar blue dye conversion by macrophages exposed to GO remains unclear, but could potentially be explained by the induction of unrelated enzymatic activities. Furthermore, a dose-dependent loss of cell viability was observed for FLG at 24 h, with 60% viability at the highest concentration (75 μg/ml) (Fig 3C).

**Fig 4:**
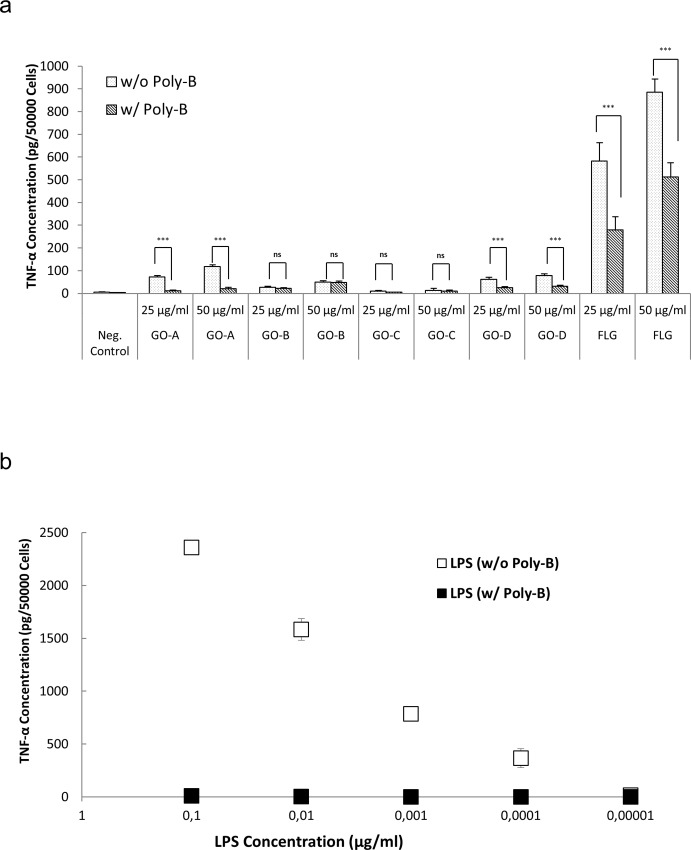
TNF-α Expression Test (TET) for endotoxin detection. a). The TET was performed to detect endotoxin content in all GBMs upon exposure of HMDM to 25 and 50 μg/ml for 24 h. The differences in TNF-α expression in the presence and absence of polymyxin B sulfate (Poly-B) (10 μM) provided evidence of the presence of endotoxin in the GO-A, GO-D and FLG samples. Such differences were not observed upon exposure of cells to GO-B and GO-C. One-way Anova with post hoc Turkey’s test was performed to analyze the statistical significance between the sample exposed with and without Poly-B. Note that also triggered significant production of TNF-α in presence of Poly-B. b) Standard curve showing relationship between LPS and TNF-α expression. Poly-B blocked LPS-triggered TNF-α production, as expected. Experiments were conducted using cells from at least three independent donors per experiment. (* = p ≤ 0.05, ** = p ≤ 0.01, *** = p ≤ 0.001).

Based on the results of the Alamar Blue assay, we selected 25 and 50 μg/ml for endotoxin evaluation by TET. The TET was performed with GBMs in the presence or absence of the endotoxin inhibitor polymyxin B sulfate [25] and LPS was included as a positive control. If HMDMs exposed to GO produce TNF-α and if the levels of TNF-α are equivalent in the presence or absence of polymyxin B sulfate, then TNF-α production is an intrinsic feature of the GBM. While if HMDM express less TNF-α upon exposure to GBM in the presence of polymyxin B sulfate then the GBM is endotoxin contaminated. If there is no secretion of TNF-α, then there is no endotoxin present (Fig 1). HMDM were thus exposed for 24 h to 25 and 50 μg/ml of GBMs in the presence or absence of 10 μM polymyxin B sulfate. Then, supernatants were collected and TNF-α was measured using a specific ELISA. In addition, HMDM were exposed to different doses of LPS (100 ng/ml to 10 pg/ml) to generate a standard curve. The TET showed that GO-A triggered a moderate, albeit significant production of TNF-α in macrophages which was suppressed in the presence of polymyxin B, indicating that GO-A was, in fact, endotoxin-contaminated (Fig 4A). Based on the standard curve shown in Fig 4B, 50 μg/ml GO-A is concluded to contain 30 pg/ml LPS. The results for GO-B are discussed below. The commercial GO-C sample did not trigger TNF-α production in macrophages at 25 or 50 μg/ml (with or without polymyxin B). On the other hand, the commercial GO-D sample triggered a minor, but statistically significant production of TNF-α which was suppressed in the presence of polymyxin B, indicating that this sample contained endotoxin. Finally, FLG, triggered significant TNF-α production both in the presence and absence of polymyxin B, although in the presence of the endotoxin inhibitor, the level of TNF-α production was substantially reduced (Fig 4A). This result clearly indicates a) that FLG was endotoxin contaminated, and b) that FLG has an inherent propensity to trigger pro-inflammatory cytokine production. Thus, even in the presence of polymyxin B, some GBMs (eg., GO-A and GO-D) induced a low, but detectable level of TNF-α production while a significant level of TNF-α production was noted for FLG. Hence, the TET assay, conducted in the absence or presence of an endotoxin inhibitor to exclude endotoxin mediated effects, has revealed the intrinsic pro-inflammatory properties of certain GBMs. Qu et al. reported previously that GO induced necrotic cell death in murine macrophages and this was suggested to be mediated through autocrine TNF-α signaling in these cells [19]. The authors also argued that the effects of GO were mediated by activation of TLR4, a pattern recognition receptor that serves as a key sensor of endotoxin. However, it should be noted that endotoxin contamination of the test materials could yield ambiguous results and experiments conducted with or without polymyxin B could help to address this. Furthermore, although GO-A and GO-D induced statistically significant levels of TNF-α production in the presence of polymyxin B, the TNF-α levels remained very low (below 50 pg/50.000 cells) (Fig 4A). GO-C, on the other hand, did not induce any TNF-α secretion. Comparing the lateral dimensions of the GO samples (Table 1), it appears that large flakes (i.e., GO-A and GO-D) are capable of inducing low, but statistically significant TNF-α production, while small flakes (GO-C) did not elicit such effects. This result is in accordance with previous studies that have pointed to a crucial role of the lateral dimensions of GO in activating macrophages and stimulating pro-inflammatory effects [29]. FLG induced marked TNF-α production in the presence of polymyxin B (about 500 pg/50.000 cells) (Fig 4A) and induced dose-dependent cytotoxicity (Fig 3C) while the GO samples were non-cytotoxic, suggesting, overall, that GO is less toxic than FLG.

### Endotoxin-free production of GO

**Fig 5:**
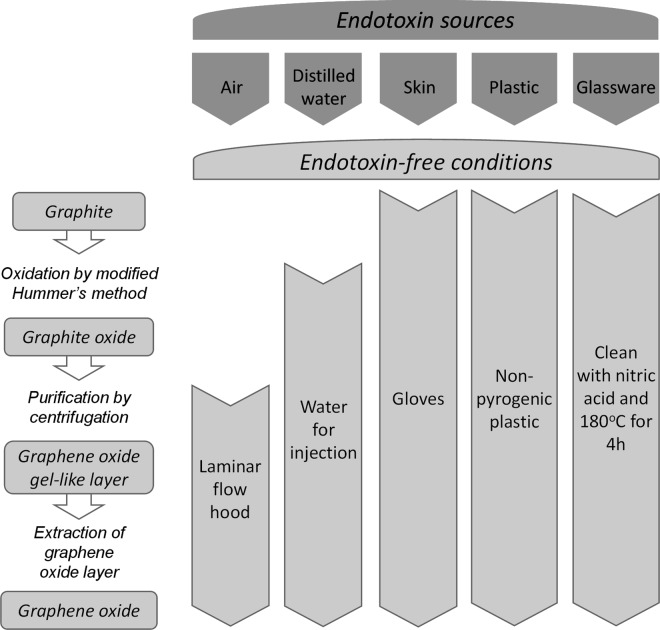
Flow chart depicting the main endotoxin sources identified in the preparation of GO by Hummer’s method and guidelines for endotoxin-free conditions for the production of GO samples.

Next, in an attempt to produce endotoxin-free GO, the following precautions were implemented. First, the main endotoxin sources involved in the preparation of GO by the modified Hummer’s method were identified as follows: air, distilled water (this is the main source of endotoxin contamination), skin, plastic containers and glassware. Due to the high thermal stability of endotoxins, they are difficult to eliminate under regular sterilizing conditions [30]. For the production of GO by the modified Hummer’s method, the highly acidic environment combined with high temperatures (100°C) may likely eliminate endotoxins from all materials and reagents involved in the oxidation step from graphite to graphite oxide. Moreover, to minimize endotoxin contamination, glassware was cleaned with 50% nitric acid and de-pyrogenated at 180°C for 4 h in the oven, before the preparation of GO. Also, gloves were used in the entire process to minimize endotoxin contamination from the skin. Moreover, to avoid contamination from the air, the preparation of GO was carried out in the laminar flow hood. All plastic ware involved in the purification and extraction of the GO was non-pyrogenic. Distilled water, the most common source of endotoxin contamination, was replaced with endotoxin-free water. By keeping the entire preparation of GO under sterile conditions, we produced the sample designated as GO-B. The flowchart for the modified, endotoxin-free production of GO by Hummer’s method is shown in Fig 5.

Following this protocol, detailed physicochemical characterization was performed on the GO-B sample to verify that the procedure did not alter the physicochemical properties of this material (Table 1). As previously shown for GO-A, the GO-B sample was non-cytotoxic for primary human macrophages (Fig 3C). Moreover, as already noted, both GO-A and GO-B yielded ambiguous results when the LAL assay was applied. Therefore, we proceeded to assess the GO-B sample for endotoxin using the TET. The results showed that TNF-α levels produced by HMDM upon exposure to GO-B at 25 and 50 μg/ml for 24 h were considerably lower, as compared to the TNF-α levels generated by GO-A; moreover, the TNF-α secretion was unaffected by polymyxin B sulfate, demonstrating that this effect was not due to endotoxin contamination of the sample (Fig 4A). Hence, with these results, we could validate the revised guidelines for sterile production of GO (Fig 5), and we could also show that GO triggers a low level of TNF-α production in macrophages, in the absence of any cytotoxicity. Based on the standard curve (Fig 4B), we may conclude that 50 μg/ml GO-B contained approx. 0.5 pg/ml LPS, well below the FDA-mandated level for medical devices.

### Considerations on assay sensitivity

It has been reported that the MAT has a sensitivity of 50 pg/ ml LPS [31]. Notably, it has also been reported that LPS at a concentration of 10 pg/ml can stimulate PBMCs to produce TNF-α [32], suggesting that it is of considerable importance, when assessing for effects on immune-competent cells, to be able to detect very low levels of endotoxin contamination. The data presented herein indicated that the TET displays a high sensitivity for endotoxin. Different endotoxins have different potencies, but an equivalency of 100 pg = 1 EU is commonly assumed to convert endotoxin mass to activity [27]. Therefore, by converting the mass unit to potency, it can be argued that 50 μg/ml of GO-A and GO-B contain 0.3 EU/ml and approx. 0.005 EU/ml endotoxin, respectively, according to our TET assay results (Fig 4A and 4B). Hence, the TET has a sensitivity comparable to the chromogenic LAL assay (0.001 EU/ml), but as shown here it does not suffer from interference of the test material. Therefore, we propose that the TET is well suited as an endotoxin detection assay for GBMs, and other materials, that may display interference with conventional LAL assays. In general, interference with the LAL assay may be reduced by diluting the sample, but this would also reduce the capacity of the assay to detect endotoxin contamination in the sample. Finally, it is noted that the TET is only suitable for materials with low cytotoxicity towards primary macrophages. In the present study, all GO samples tested were found to be completely non-cytotoxic at the doses tested (up to 75 μg/ml) while FLG displayed a dose-dependent cytotoxicity for macrophages, but a non-cytotoxic dose could be established for all the materials, for use in the TET.

## Conclusions

Nanomaterials, not least carbon-based nanomaterials, have been shown to interfere with commonly used assays for cytotoxicity or endotoxin evaluation, leading to erroneous results in toxicological testing of such materials [33–35]. In the present study, GO was found to cause significant interference in the LAL assay used for endotoxin detection, and ambiguous results were obtained using different LAL assay formats. The reasons for the interference with the chromogenic LAL assay remain to be understood, but this can possibly be explained by the overlap in absorbance at 405 nm wavelength between GO and the substrate indicator (i.e., p-nitroaniline) detected in the LAL assay. The problem was overcome by developing a functional assay, the TNF-α expression test or TET, based on the detection of TNF-α secretion by primary human macrophages exposed to GO in the presence or absence of polymyxin B sulfate, a specific LPS inhibitor. It is important that non-cytotoxic concentrations of the test material are applied when assessing for endotoxin contamination. During the course of these studies, we have shown that GO samples are non-cytotoxic for primary human macrophages while FLG displayed a dose-dependent cytotoxicity. Moreover, the TET revealed that GO from various sources triggers negligible TNF-α production in primary human macrophages, while FLG elicits a strong pro-inflammatory response, irrespective of endotoxin contamination. It is suggested that the TET is a suitable method for endotoxin detection in GBMs, as well as to assess their intrinsic inflammogenic properties. Finally, supported by this assay, we have provided simple guidelines for the endotoxin-free production of GO.

## Abbreviations

CSE: control standard endotoxin
GBM: graphene based materials
GO: graphene oxide
FLG: few- layered graphene
HMDM: human monocyte-derived macrophages
LAL: limulus amebocyte lysate
LPS: lipopolysaccharide
MAT: monocyte activation test
PBMC: peripheral blood mononuclear cells
TET: TNF-α expression test
TLR: Toll-like receptor
TNF: tumor necrosis factor
